# Activation instead of blocking mesolimbic dopaminergic reward circuitry is a preferred modality in the long term treatment of reward deficiency syndrome (RDS): a commentary

**DOI:** 10.1186/1742-4682-5-24

**Published:** 2008-11-12

**Authors:** Kenneth Blum, Amanda Lih Chuan Chen, Thomas JH Chen, Eric R Braverman, Jeffrey Reinking, Seth H Blum, Kimberly Cassel, Bernard W Downs, Roger L Waite, Lonna Williams, Thomas J Prihoda, Mallory M Kerner, Tomas Palomo, David E Comings, Howard Tung, Patrick Rhoades, Marlene Oscar-Berman

**Affiliations:** 1 Department of Physiology & Pharmacology, Wake Forest University School of Medicine, Winston-Salem, NC, USA; 2 Engineering & Management of Advanced Technology, Chang Jung University, Taiwan, PR China; 3 Department of Occupational Health and Safety, Chang Jung University, Taiwan, PR China; 4 Department of Neurosurgery, Weill Cornell College of Medicine, New York, NY, USA; 5 Department of Occupational Health and Safety, Chang Jung University, Taiwan, PR China; 6 Department of Psychoneurogenetics, Synaptamine™, Inc., San Antonio, TX, USA; 7 Deparment of Nutrigenomics, LifeGen, Inc, La Jolla, CA, USA; 8 Department of Pathology, University of Texas Health Science Center, San Antonio, TX, USA; 9 Department of Neurological Research, Path Research Foundation, New York, NY, USA; 10 Hospital Universitario 12 de Octubre, Madrid, Spain; 11 Carlsbad Science Foundation, Emeritus, City Of Hope National Medical Center, Duarte, CA, USA; 12 University of California, San Diego Medical Center, Neurological Surgery (Brain and spinal disorders), San Diego, CA, USA; 13 Central Valley Pain Management & Wellness Modesto, CA, USA; 14 Boston University School of Medicine and Boston VAMC, Boston, MA, USA

## Abstract

**Background and hypothesis:**

Based on neurochemical and genetic evidence, we suggest that both prevention and treatment of multiple addictions, such as dependence to alcohol, nicotine and glucose, should involve a biphasic approach. Thus, acute treatment should consist of preferential blocking of postsynaptic Nucleus Accumbens (NAc) dopamine receptors (D1-D5), whereas long term activation of the mesolimbic dopaminergic system should involve activation and/or release of Dopamine (DA) at the NAc site. Failure to do so will result in abnormal mood, behavior and potential suicide ideation. Individuals possessing a paucity of serotonergic and/or dopaminergic receptors, and an increased rate of synaptic DA catabolism due to high catabolic genotype of the COMT gene, are predisposed to self-medicating any substance or behavior that will activate DA release, including alcohol, opiates, psychostimulants, nicotine, gambling, sex, and even excessive internet gaming. Acute utilization of these substances and/or stimulatory behaviors induces a feeling of well being. Unfortunately, sustained and prolonged abuse leads to a toxic" pseudo feeling" of well being resulting in tolerance and disease or discomfort. Thus, a reduced number of DA receptors, due to carrying the DRD2 A1 allelic genotype, results in excessive craving behavior; whereas a normal or sufficient amount of DA receptors results in low craving behavior. In terms of preventing substance abuse, one goal would be to induce a proliferation of DA D2 receptors in genetically prone individuals. While in vivo experiments using a typical D2 receptor agonist induce down regulation, experiments in vitro have shown that constant stimulation of the DA receptor system via a known D2 agonist results in significant proliferation of D2 receptors in spite of genetic antecedents. In essence, D2 receptor stimulation signals negative feedback mechanisms in the mesolimbic system to induce mRNA expression causing proliferation of D2 receptors.

**Proposal and conclusion:**

The authors propose that D2 receptor stimulation can be accomplished via the use of Synapatmine™, a natural but therapeutic nutraceutical formulation that potentially induces DA release, causing the same induction of D2-directed mRNA and thus proliferation of D2 receptors in the human. This proliferation of D2 receptors in turn will induce the attenuation of craving behavior. In fact as mentioned earlier, this model has been proven in research showing DNA-directed compensatory overexpression (a form of gene therapy) of the DRD2 receptors, resulting in a significant reduction in alcohol craving behavior in alcohol preferring rodents. Utilizing natural dopaminergic repletion therapy to promote long term dopaminergic activation will ultimately lead to a common, safe and effective modality to treat Reward Deficiency Syndrome (RDS) behaviors including Substance Use Disorders (SUD), Attention Deficit Hyperactivity Disorder (ADHD), Obesity and other reward deficient aberrant behaviors. This concept is further supported by the more comprehensive understanding of the role of dopamine in the NAc as a "wanting" messenger in the meso-limbic DA system.

## Background

It is well known that brain reward circuitry is regulated by neurotransmitter interactions and net release of the substance Dopamine (DA) in the Nucleus accumbens (NAc) [1]. The major loci for feelings of well-being and reward occur in the meso-limbic system of the brain. The natural sequence of events of the "brain reward cascade" leading to reward involves the inter-relationship of at least four important neurochemical pathways: serotonergic (5-HT); enkephalinergic (Enk), GABAergic (GABA), and dopaminergic (DA). The synthesis, vesicle storage, metabolism, release and function of these neurotransmitters are regulated by genes and the expression thereof in terms of messenger RNA (mRNA) directed proteins. It has been postulated that genome orientated research will provide genetic testing that will categorize individuals as to their specific neurochemical makeup and thus provide useful information to assist in appropriate development of the most correct treatment options for the patient requiring psychiatric care [2]. DA is a substance with many important neurochemical functions and has been credited with resultant behavioral effects such as "pleasure," "stress reduction" and "wanting". Simply stated, without the normal functionality of DA, an individual will be lacking hedonic response and an inability to cope with stress [3]. Thus genetic hypodopaminergic activity of the brain predisposes an individual to seek substances and/or behaviors that will overcome this anhedonic state by activating meso-limbic dopaminergic centers [4]. It turns out that these substances and behaviors include: alcohol, opiates, psychostimulants, nicotine, carbohydrates, cannabinoids, gambling, sex, and indulgence in any excessive pleasure or thrill seeking behaviors, like video gaming etc. [5-16]. Use of these substances and engaging in these aforementioned behaviors commonly induces the release of neuronal DA into the synapse at the NAc, the reward center of the brain [3]. Acute indulgence in these behaviors can be classified as self-medicating and leads to a preferential release of DA, which overcomes the hypodopaminergic state for that individual. The resultant self-medication provides a temporary relief of discomfort and a "pseudo feeling" of well-being [17]. Unfortunately, chronic abuse of these psychoactive substances and excessive indulgence in the aberrant behaviors leads to inactivation of the brain reward cascade (*i.e*. neurotransmitter synthesis inhibition, neurotransmitter storage depletion, toxic formation of pseudo neurotransmitters and receptor dysfunction (structural and or density)). The abusive behaviors also lead to neurotransmitter dysfunction via depletion. Therefore both substance seeking and pathological behaviors as ways of providing a feel good response (FGR) "fix" result in ever escalating and uncontrollable craving behavior. It has been well established that individuals possessing certain genetic polymorphisms (variations) are particularly prone to amplified polymorphic expressions with environmental or lifestyle insult and will be at increased risk for impulsive, compulsive and addictive behaviors [18]. Such common genetic antecedents influencing the natural brain reward cascade provide the understanding that impulsive, compulsive and addictive behaviors are commonly linked and support the emerging concept of Reward Deficiency Syndrome (RDS) as an umbrella term to characterize and classify these commonly linked genetically induced behaviors [19-21]. In this scenario any and all of these abusable psychoactive drugs or pathological behaviors are candidates for addiction (tolerance/dependence) and are chosen by the individual as a function of both genes and environmental factors (*e.g*. availability, peer pressure, etc.) [18].

## Brain reward cascade explanation

**Figure 1:**
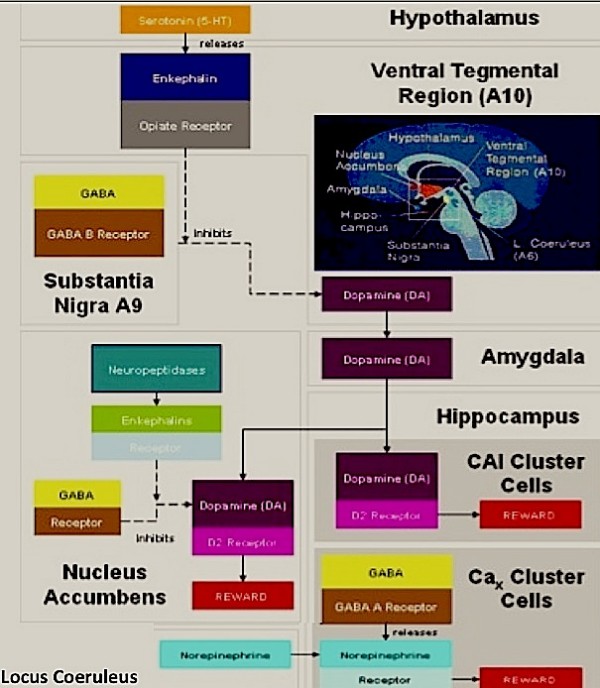
**Brain reward cascade **[1]**-modified with permission from *Gene Therapy Press***. In this cascade, stimulation of the serotonergic system in the hypothalamus leads to the stimulation of delta/mu receptors by serotonin to cause a release of enkephalins. Activation of the enkephalinergic system induces an inhibition of GABA transmission at the substantia nigra by enkephalin stimulation of mu receptors at GABA neurons. This inhibitory effect allows for the fine tuning of GABA activity. This provides the normal release of dopamine at the projected area of the n. accumbens (reward site of the brain). It is noteworthy that other important neurotransmitters and receptors are involved such as endocannibinoids and glutamate.

While dopamine (DA) is critical to maintain normalization of natural rewards, the neuronal release of DA into NAc synaptic sites is somewhat complex. In 1989 our laboratory proposed an interactive cascade of events of mesolimbic function that lead to net DA release [1]. It was termed the "brain reward cascade' (see Figure 1)

The interactions of activities in the separate subsystems mentioned above merge together into the much larger global system. These activities take place simultaneously and in a specific sequence, merging like a cascade. The end result is a sense of peace, pleasure, and well-being when these systems work normally. If there is a deficiency or imbalance, the system works abnormally, causing the sense of well-being to be displaced by feelings of anxiety, anger, low self-esteem, and/or other "bad feelings"[15]. This can lead to cravings for substances and/or behaviors that mask or relieve those bad feelings such as carbohydrate bingeing, alcohol, or cocaine; or to other addictive behaviors such as compulsive gambling, compulsive sex, workaholism, or engaging in high risk activities; all excessive desires spurred by the need for a dopamine fix [19-21].

Other research has confirmed that the reward sensation is related to complex cascade reactions involving several neurotransmitters and structures in the limbic system [22]. The ultimate result of the process is the activation of the meso-limbic dopamine pathway, which starts in the tegmental ventral area and ends at the dopamine D2 receptors on the cell membranes of neurons located in the NAc and the hippocampus [22].

The process, as described by Blum and Kozlowski [1], starts in the hypothalamus with the excitatory activity of serotonin-releasing neurons. This causes the release of the opioid peptide met-enkephalin in the ventral tegmental area, which inhibits the activity of neurons that release the inhibitory neurotransmitter gamma-aminobutyric acid (GABA). The disinhibition of dopamine-containing neurons in the ventral tegmental area (VTA) allows them to release dopamine in the NAc and (via amygdala) in certain parts of the hippocampus, permitting the completion of the cascade and the development of the reward sensation [23]. Usually, if the cascade is working properly, the reward or feeling of "well-being", or FGR, is obtained provided certain basic conditions are fulfilled [1].

## RDS and genetic antecedents

Understanding the brain reward cascade provides insight into the development of a blue-print for unlocking certain candidate genes and polymorphisms that could impact the brain in a negative manor. Impairment of the brain reward cascade ultimately leads to a reduction of net DA release, a reduction in dopamine receptors and as such an enhancement of substance craving activity. While there are many genes involved, it has been adequately established that polymorphisms of the serotonergic- 2 A receptor (5-HTT2a); dopamine D2 receptor (DRD2) and the Catechol-o-methyl-transferase (COMT) genes predispose individuals to aberrant RDS behaviors especially cravings [19,71]. In the case of both serotonin and dopamine gene polymorphisms, their respective receptors are significantly lower than normal [24,25]. A certain type of polymorphism in the COMT gene results in an increase in the catabolism of synaptic DA and subsequent reduced function [26]. Polymorphic identification of at least these three genes provides insight into a genetic window of an impaired brain reward cascade that places that individual at high risk for excessive craving behaviors. Based on a published [27] mathematical "Bayesian" approach, it was found that individuals carrying these known polymorphisms (in particular the DRD2) have a 74% chance that given the trigger of environmental insult will develop RDS (for reviews see [1,18-21]).

## Role of dopamine agonists in proliferation of D2 receptors

Studies in vitro have shown that constant stimulation of DA receptors by agonists result in proliferation of Dopamine D2 receptors coupled to G proteins. Specifically it was shown [28,29] in transfected kidney cells and expressed in *Spodoptera frugiperda *insect cells that stimulation of DA receptors by the pure D2 receptor agonist Bromocriptine resulted in proliferation of D2 receptors over a 14 day period. In the same study it was shown that administration of a DA antagonist caused the proliferation of D2 antagonist receptors as well. These two independent effects suggest that environmental manipulation in spite of genetic antecedents will result in receptor proliferation. This can best be explained by the understanding that agonist activity involves the stimulation of the mRNA that is involved in transcription. Activation of the DRD2/mRNA results in a negative feedback that promotes an enhancement of mRNA directed D2 receptor proliferation. This fact becomes very important when coupled with the findings that an increase in substance seeking is due to a paucity of DA D2 receptors [24,25]. Therefore, if low D2 receptors equate to increased craving behavior then an increase in D2 receptors should result in attenuation of craving behavior. Our solution is to stimulate DA release at the NAc naturally, not via powerful DA agonists that could ultimately lead to DA down-regulation. Whereas DA activation could occur with targeted pharmaceuticals such as Bromocriptine or other DA agonists [30], we prefer a more natural approach developed to mimic the brain reward cascade; in essence, through the utilization of precursor amino-acids and simultaneous enkephalinase/COMT inhibition, which we suggest will systematically induce natural release of DA without side effects.

## Traditional anti-craving treatments block dopamine activity at the brain reward centers

Most recent examples of pharmaceuticals that block DA release and or receptor activation include Acomplia (Rimonabant), the cannabinoid (CB1) receptor blocker and possibly Gabapentin. While there are numerous studies supporting the therapeutic benefits of Acomplia as an anti-craving drug the long term adverse effects resulted in a recent rejection by the United States Federal Drug Administration (FDA). A recent PUBMED search revealed 1007 papers on Acomplia. Since the prevalence of obesity continues to increase, there is a demand for effective and safe anti-obesity agents that can produce and maintain weight loss and improve comorbidity. Christensen et al [31] did a meta-analysis of all published randomized controlled trials to assess the efficacy and safety of the newly approved anti-obesity agent Rimonabant. They searched the Cochrane database and Controlled Trials Register, Medline via Pubmed, Embase via WebSpirs, Web of Science, Scopus, and reference lists up to July, 2007. They collected data from four double-blind, randomized controlled trials (including 4105 participants) that compared 20 mg per day Rimonabant with placebo. Patients given Rimonabant had a 4.7 kg (95% CI 4.1–5.3 kg; p < 0.0001) greater weight reduction after 1 year than did those given placebo. Rimonabant caused significantly more adverse events than did placebo (Odds Ratio (OR) = 1.4; p = 0.0007; number needed to harm = 25 individuals [95% CI 17–58]), and 1.4 times more serious adverse events (OR = 1.4; p = 0.03; number needed to harm = 59 [27–830]). Patients given Rimonabant were 2.5 times more likely to discontinue the treatment because of depressive mood disorders than were those given placebo (OR = 2.5; p = 0.01; number needed to harm = 49). Furthermore, anxiety caused more patients to discontinue treatment in Rimonabant groups than in placebo groups (OR = 3.0; p = 0.03; number needed to harm = 166). Their findings suggest that 20 mg per day of Rimonabant increases the risk of adverse psychiatric events – i.e. depressed mood disorders and anxiety; despite depressed mood being an exclusion criterion in these trials. Taken together with the recent US Food and Drug Administration finding of increased risk of suicide during treatment with Rimonabant, these researchers recommend increased alertness by physicians to these potentially severe psychiatric adverse reactions. Concerning this report, we propose that the negative effects on mood are due to the continued blockade of naturally required DA release at the NAc.

Gabapentin is a gamma-aminobutyric acid (GABA) analogue, with GABAmimetic pharmacological properties. Gabapentin is used for the treatment of seizures, anxiety and neuropathic pain. It has been proposed that Gabapentin may be useful in the treatment of cocaine dependence. However, clinical trials with Gabapentin have shown conflicting results, while preclinical studies are sparse. In one study, Peng et al [32] investigated the effects of Gabapentin on intravenous cocaine self-administration and cocaine-triggered reinstatement of drug-seeking behavior, as well as on cocaine-enhanced DA in the NAc. They found that Gabapentin (25–200 mg/kg, i.p., 30 min or 2 h prior to cocaine) failed to inhibit intravenous cocaine (0.5 mg/kg/infusion) self-administration under a fixed-ratio reinforcement schedule or cocaine-triggered reinstatement of cocaine-seeking behavior. In vivo microdialysis showed that the same doses of Gabapentin produced a modest increase (approximately 50%, p < 0.05) in extracellular NAc GABA levels, but failed to alter either basal or cocaine-enhanced NAc DA. These data suggest that Gabapentin is a weak GABA-mimic drug. At the doses tested, it has no effect in the addiction-related animal behavioral models. This is in striking contrast to positive findings in the same animal models shown by another GABAmimetic – gamma-vinyl GABA – by Garner's group (see [18] for review). Based on our current theoretical model we are opposed to the use of Gabapentin to treat substance seeking behavior especially in long term care.

Other than a few scientific groups that suggest serotonergic/dopaminergic agonist therapy [33], most strategies embrace dopaminergic receptor blockade/attenuation of dopamine release [2,3,18-21]. We propose that, in most circumstances, utilization of amino acid precursors affecting positive dopaminergic activation is a better alternative [34-48] (see tables 1 &2).

**Table 1:** Summary of completed clinical studies with nutraceutical supplementation: a literature review

Drug Abused or Dysfunction	Supplement Used	No. of Patients	No. of Days	Study Type	Significant Results	Publication
Alcohol	SAAVE	22	28	TO IP	100% decrease in BUD scores. Detoxification measures: reduction in benzodiazepine requirement, reduction in withdrawal tremors after 72 hours, reduction in depression	Blum K, Trachtenberg MC, Ramsey J. Improvement of inpatient treatment of the alcoholic as a function of neuronutrient restoration: a pilot study. *Int J Addiction*. 1988; 23:991–98.

						Blum K, Trachtenberg MC. Neurogenic deficits caused by alcoholism: restoration by SAAVE. *Journal of Psychoactive Drugs*. 1988; 20:297.

Alcohol plus Polydrugs	SAAVE	62	21	DBPC IP	Reduction in psychosocial stress reduction as measured by SCL, reduced BESS score, improved physical score, six-fold decrease in likelihood of leaving AMA after five days.	Blum et al. Enkephalinase inhibition and precursor amino acid loading improves inpatient treatment of alcoholics and poly-drug abusers: a double-blind placebo-controlled study of the neuronutrient intervention adjunct SAAVE. *Alcohol*. 1989; 5:481.

Cocaine	Tropamine	54	30	TO IP	Drug hunger significantly reduced in patients taking SAAVE as compared to controls; 4.2 percent AMA rate for patients on Tropamine versus 28 percent for patients on SAAVE and 37 percent for controls. </SPAN>	Blum et al. Reduction of both drug hunger and withdrawal against advice rate of cocaine abusers in a 30 day inpatient treatment program with the neuronutrient tropamine. *Curr Ther Res*. 1988; 43:1204.

Alcohol and Cocaine	SAAVE and Tropamine	60	379	TO CP	At end of one year over 50 percent of the alcoholic DUI offenders not using SAAVE dropped out of the program while less than 15 percent of those using SAAVE dropped out. For the cocaine abusers over 90 percent of the Non-Tropamaine group dropped out, but less than 25 percent of the patients in the control group.	Brown et al. Neurodynamics of relapse prevention: a neuronutrient approach to outpatient DUI offenders. *J. Psychiatric Drugs*. 1990; 22:173.

Over-Eating	PCAL 103	27	90	TO OP	The PCAL 103 group lost an average of 27 pounds in 90 days compared with an average loss of 10 pounds for the control group. Only 18.2 percent of the PCAL 103 patient group relapsed compared to 82 percent of the patients in the control group.	Blum et al.20 Neuronutrient effects on weight loss on carbohydrate bingeing in a bariatric setting. *Curr Ther Res*. 1990; 48:2a17.

Over-Eating	PCAL 103	247	730	PCOT OP	After two years, craving and binge eating were reduced one-third in group of patients on PCAL 103, as compared to the control patients. PCAL 103 group regained 14.7 pounds of their lost weight compared with 41.7 percent weight regained in control patients.	Blum K, Cull JG, Chen JHT, Garcia-Swan S, Holder JM, Wood R, et al. Clinical relevance of PhenCal in maintaining weight loss in an open-label, controlled 2-year study. *Curr Ther Res*. 1997; 58:745–63.

Over-Eating	Chromium Picolinate (CP) and L-Camitine	40	112	RDBPC CP	21 percent increase (p < 0.001) in resting metabolic rate (RMR), no change in lean body mass (LBM), RMR:LBM increased 25 percent (p < 0.001). Body fat decreased approximately 1.5 lbs./week, and reduction in serum cholesterol while incre asing RMR with no loss of LBM	Kaats FE et al. The short-term therapeutic effect of treating obesity with a plan of improved nutrition and moderate caloric restriction. *Curr Ther Res*. 1992; 51:261.

Over-Eating	Chromium Picolinate	32	180	DBPC OP	After six months the CrP group had an increase in lean body mass and avoided non-fat related weight loss. Difference between groups was significant at p < 0.001.	Bahadori B, Habersack S, Schneider H, Wascher TC, Topiak H. Treatment with chromium picolinate improves lean body mass in patients following weight reduction. *Federation Am Soc Exp Bio *1995.

Over-Eating	Chromium Picolinate	154	72	RDBPC OP	200 and 400 mcg of CrP brought about significant changes in Body Mass composition indicies when compared with placebo	Kaats FE, Blum K, Fisher JA, Aldeman JA. Effects of chromium picolinate supplementation on body mass composition: a randomized, double-blind, placebo-controlled study. *Curr Ther Res*. 1996; 57:747–56

Over-Eating	Chromium Picolinate	122	90	RDBPC OP	After controlling for differences in caloric expenditure and caloric intake as compared with the placebo group, 400 mcg CrP group lost significantly more weight (p < 0.001) and body fat (p < 0.004), had a greater reduction in body fat (p < 0.001), significantly improve body composition (p < 0.004).	Kaats FE, Blum K, Pullin D, Keith SC, Wood R. A randomized double-masked placebo-controlled study of the effects of chromium picolinate supplementation on body composition: a replication of previous study. *Curr Ther Res*. 1998; 59:379–88.

Over-Eating	Chromium Picolinate	122	90	RDBPC OP	Measures of changes in fat weight, change in body weight, percent change in weight, and body weight changes in kgms were all significant in A2/A2 group, and non-significant in A1/A2 and A1/A1 carriers.	Blum K, Kaats G, Eisenbery A, Sherman M, Davis K, Comings DE, Cull JG, Ch en THJ, Wood R, Bucci L, Wise JA, Braverman ER, and Pullin D. Chromium Picolinate Induces Changes in Body Composition as a Function of the Taq1 Dopamine D2 Receptor A1 Alleles. Submitted to *International J. Eat. Dis*.

Over-Eating	Chromium Picolinate and Chromium Picolinate comparison	43	63	ROTPC OP	CrP supplementation resulted in significant weight gain, while exercise training combined with CrP supplementation resulted in significant weight loss and lowered insulin response to an oral glucose load. Concluded high levels of CrP supplementation are contraindicated for weight loss, in young obese women. Moreover, results suggested that exercise combined with CrP supplementation may be more beneficial than exercise training alone for modification of certain CAD or NIDDM risk factors	Grant KE, Chandler RM, Castle AL, Ivy JL. Chromium and exercise training: effect on obese women.20*J Am Sports Med *1997; 29(8):992–8.

Healthy Volunteers	Tropagen	15	30	DBPC OP	Non-drug subjects with Tropagen performed better on computer memory and performance tasks as measured with P300 wave evoked potential. Changes in P300 wave evoked potential result in better focusing ADHD patients	Defrance JJ, Hymel C, Trachtenberg MC et al. Enhancement of attention processing by Kantrol in healthy humans: A pilot study. *Clin Electroencephalgr*. 1997; 28:68–75.

Abbreviations used: BUD – building up to drink; AMA – withdrawal against medical advice; OP – outpatient; MMPI – Minnesota Multiphasic personality inventory; DB – double-blind; IP – inpatient; SCL – skin conductance level; BESS – behavioral, emotional, social, spiritual; DBPC – double-blind placebo-controlled; DUI – driving under the influence; R – randomized; TO – open trial

***Source : Chen et al 2004 ***[39]***with permission Elsevier***.

**Table 2:** Amino acid nutrition therapy

**Supplemental Ingredient**	**Restored Brain Chemical**	**Addictive Substance Abuse**	**Amino Acid Deficiency Symptoms**	**Expected Behavior Change**
D-Phenylalanine or DL-Phenylalanine	Enkephalins, Endorphins	Heroin, Alcohol, Marijuana, Sweets, Starches, Chocolate, Tobacco	Most Reward Deficiency Syndrome (RDS) conditions sensitive to physical or emotional pain. Crave comfort and pleasure. Desire certain food or drugs. D-Phenylalaine is a known enkephalinease inhibitor.	Reward stimulation. Anti-craving. Mild anti-depression. Mild improved energy and focus. D-Phenylalaine promontes pain relief, increases pleasure.

L-Phenylalanine or L-Tyrosine	Norepinephrine, Dopamine	Caffeine, Speed, Cocaine, Marijuana, Aspartame, Chocolate, Alcohol, Tobacco, Sweets, Starches	Most RDS conditions. Depression, low energy. Lack of focus and concentration. Attention-deficit disorder.	Reward stimulation. Anti-craving. Anti-depression. Increased energy. Improved mental focus.

L-Tryptophan or 5 hydroxytryptophan (5HTP)	Serotonin	Sweets, Alcohol, Starch, Ecstasy, Marijuana, Chocolate, Tobacco	Low self esteem. Obsessive/compulsive behaviors. Irritability or rage. Sleep problems. Afternoon or evening cravings. Negativity. Heat intolerance. Fibromyalgia. Seasonal affective disorder.	Anti-craving. Anti-depression. Anti-insomnia. Improved appetite control. Improvement in all mood and other serotonin deficiency syndromes.

Gamma-amino butyric acid (GABA)	GABA	Valium, Alcohol, Marijuana, Tobacco, Sweetes, Starches	Feeling of being stressed out. Nervous. Tense muscles. Trouble relaxing.	Promotes calmness. Promotes relaxation.

L-Glutamine	GABA (mild enhancement). Fuel source for entire brain	Sweets, Starches, Alcohol	Stress. Mood swings. Hypoglycemia.	Anti-craving, anti-stress. Levels blood sugar and mood. GABA (mild enhancement). Fuel source for entire brain.

**Table 2 Comments: **Rhodiola rosea has been added to the formula and is a known Catechol-O-methyl transferase (COMT) inhibitor. This provides more synaptic dopamine in the VTA/NAc.

Source: Perfumi M, Mattioli L. Adaptogenic and central system effects of single doses of 3% rosavin and 1% salidroside Rhodiola rosea L. extract in mice. *Phytother Res*. 21 2007 37–43

Chromium salts – This has been added to the formula to enhance insulin sensitivity and resultant brain concentration of serotonin.

Note: To assist in amino acid nutritional therapy, the use of a multivitamin/mineral formula is recommended. Many vitamins and minerals serve as co-factors in neurotransmitter synthesis. They also serve to restore general balance, vitality and well-being to the RDS patient who typically is in a state of poor nutritional health. The utilization of GABA is limited due to its polar nature and ability to cross the blood brain barrier. Glutamate is used in a low level only to prevent over-inhibition of enkephalin breakdown and subsequent inhibition of GABAergic spiny neurons of the substantia nigra.

## Amino acid therapy as an anti-craving agent

Although DA release (and/or DA receptor binding) could in theory be potentiated by the above proposed ingredients (summarized in table 1) for dopaminergic activation, no one to date has actually shown this important potential and is the subject of future intensive investigation. However indirect support is derived from the effects obtained with these ingredients in a number of clinical trials over two decades (see table 1).

Table 1 illustrates the anti-craving and other effects observed with the Synaptamine™ complex. Other more recent published clinical trials include:

1. In a one year open trial consisting of 600 patients moderate to severe alcoholics utilization of both Oral and IV forms of Synaptamine resulted in significant reduction in cravings; reduced depression, reduced anxiety; reduced anger; reduced fatigue; reduced lack of energy, and reduced crisis [36].

2. In a one year open trial consisting of 76 patients severe poly drug addicts utilization of oral forms of Synaptamine resulted in significant attenuation of drug cravings; reduced relapse; reduced stress; reduced depression; reduced anger; and increased energy. The drop- out rate for alcoholics was only 7% [40].

3. In a one year cross sectional open trial study of 24 unscreened individuals utilization of oral Synaptamine variant resulted in the following benefits: stress reduction; sleep enhancement; increase in energy level; generalized well-being; reduction in cravings (sweets/carbs); improvement in mental focus/memory; improvement in blood sugar levels; reduction in food consumption; loss of inches around waist; loss of weight; reduction in blood pressure; improvement in workout performance; reduction in drug seeking behavior ; reduction in hyperactivity; reduction in cholesterol levels [37].

4. In a subset of 27 individuals out of 1000 self-identified obese subjects geneotyped for polymorphisms of the DRD2 gene and of those carrying the T*aq *A1 allele had a significant Pearson correlation with days on treatment compared to the A2 carriers. For the DRD2A1 carriers the number of days on Synaptamine Complex (variant changed according to geneotyping a total of five candidate genes) was 110 compared to only 52 days in A2 probands suggesting that DRD2 genotype can predict treatment compliance [76].

5. In a subset of 27 individuals out of 1000 self-identified obese subjects geneotyped for polymorphisms of the DRD2, PPAR gamma 2, MTHDFR, 5-HT2a genes and subsequently provided a customized Synaptamine variant based on polymorphisms the following significant results were obtained: weight loss; sugar craving reduction; appetite suppression; snack reduction; reduction in late night bingeing; increased perception of over-eating; increased energy; enhanced quality of sleep; and increased happiness [77].

Table 2 provides a list of proposed ingredients for dopaminergic activation.

The result of utilizing this natural dopaminergic activating approach over time should lead to neuronal DA release at the NAc, potentiating a proliferation of D2 receptors [28,29]. Moreover, support in humans is derived from anti-craving effects observed in numerous peer reviewed published clinical trials including randomized double-blind placebo controlled studies [34-48] (see also Table 2). It is noteworthy that animal gene therapy utilizing cDNA vectors of the DRD2gene implanted into the NAc results in decrease alcohol craving behavior [49]. We are cognizant that the dopaminergic activation approach should be utilized to treat not only alcohol, cocaine and nicotine cravings, but glucose craving as well. Thus the coupling of genetic antecedents and nutrition may be a very viable alternative approach for the treatment of obesity.

## Nutrigenomics of obesity: a case study

Obesity-related medical conditions are the second leading cause of death in the U.S. Classified as a chronic disease in 1985, the understanding of obesity and its causes and effects has been further elucidated through additional research into the genetic and biologic factors influencing this deadly disease. What used to be understood as primarily a behavioral problem of overeating and under-exercising has only contributed to continued increases in the rates of obesity despite increases in dieting, exercise and the understanding of genes [50]. Successful strategies to induce sustainable fat loss and manage obesity effectively have been elusive. For the most part, the tactics employed have not been multi-faceted, multi-system approaches, but have been characterized by one-dimensional metabolic approaches (e.g. cannabinoid (CB1) receptor blockade; serotonin receptor stimulation) targeted at achieving weight loss as measured by linear criteria (i.e. scale weight, Body Mass Index (BMI), percent body fat, etc).

Recent evidence indicates a much more complex and multidimensional syndrome, characterized by the simultaneous breakdown of many facets of metabolism exacerbated or limited by the predispositions of inherited genetic traits [51,52]. There is significant evidence to substantiate the existence of RDS as a new paradigm shift in the understanding of Obesity [53]. Specifically, there are genetic links to the various roles of catecholaminergic-influenced pathways in aberrant substance seeking behavior, in particular cravings for carbohydrates [14,50,53,54]. We propose that these various neurological factors involved in the etiology of obesity, regulated by genetic predispositions, are a subtype of RDS. The treatment of obesity and or metabolic syndrome genomic mechanisms may pave the way for novel prescription pharmaceuticals as well as nutritional and/or nutraceutical therapies. There is growing evidence to support the augmentation of precursor amino acid therapy and enkephalinase and COMT inhibition leading to enhanced levels of neurotransmitters: serotonin, enkephalins, GABA and dopamine/norepinephrine [26]. Utilizing the combination of nutraceuticals directed at replenishing the nutrigenomic needs of multiple pathways, including brain reward/metabolic targets, mechanistically mimicking the brain reward cascade as well as fat regulation and cell repair (DRD2, 5-HTT2a. PPAR-Gamma, MTHFR and Leptin genes) will provide significant anti-obesity benefits [1,19,20,22,34,35].

Our laboratory recently presented evidence to support the significant benefits of a DNA-directed personalized weight management solution ([34,35]; see table 2). We are proposing potential mechanisms herein, along with the rationale for utilizing this multifaceted approach to attenuate the pleiotropic defaults in obesity as well as other addictions including alcohol, cocaine and nicotine. In this regard, preliminary testing for the first time seems to support a combination of neurotransmitter precursor amino acids, enkephalinase inhibition, and catecholamine 0-methyl-transferase (C.O.M.T.) inhibition therapy. Components of a nutrigenomic formula are modified based on the identification of specific gene polymorphisms resulting from genomic testing and the determination of correct dosage levels to promote successful and sustainable results in improved body recomposition [55,56].

In summary, the impact of biomics technology and the DNA directed nutraceutical targeting of the brain reward circuitry may provide a customized approach to prevent and treat high risk individuals who are carriers of a genetic predisposition to obesity and related RDS behaviors. While over 600 genes have been associated with obesity, we believe that selective candidate genes could provide useful information. Thus, we present the necessity of exploiting systems biology and "omics" [34].

## Relapse in addiction: anti-reward

"A neurobiological model of the brain emotional systems has been proposed to explain the persistent changes in motivation that are associated with vulnerability to relapse in addiction, and this model may generalize to other psychopathology associated with dysregulated motivational systems" [57]. Addiction is conceptualized as a cycle of decreased function of brain reward systems and recruitment of antireward systems that progressively worsen, resulting in the compulsive use of drugs. This concept is similar to our concept of RDS which is counter to the normal homeostatic limitation of reward function. According to Koob and La Moal [57] "counteradaptive processes, such as opponent processes that are part of the normal homeostatic limitation of reward function, fail to return within the normal homeostatic range and are hypothesized to repeatedly drive the allostatic state. Excessive drug taking thus results in not only the short-term amelioration of the reward deficit but also suppression of the antireward system. However, in the long term, there is worsening of the underlying neurochemical dysregulations that ultimately form an allostatic state (decreased dopamine and opioid peptide function, increased corticotropin-releasing factor activity). This allostatic state is hypothesized to be reflected in a chronic deviation of reward set point that is fueled not only by dysregulation of reward circuits per se but also by recruitment of brain and hormonal stress responses. Vulnerability to addiction may involve genetic comorbidity (i.e. DRD2 gene A1 allele etc.) and developmental factors at the molecular, cellular, or neurocircuitry levels that sensitize the brain antireward systems."

Moreover, others have described relapse in specific terms emphasizing the importance of dopaminergic function. Volkow *et al*. [58] suggested that drug addiction is characterized by a set of recurring processes (intoxication, withdrawal, craving) that lead to the relapsing nature of the disorder. These researchers have used positron emission tomography to investigate in humans the role of dopamine (DA) and the brain circuits it regulates in these processes. They have shown that increases in DA are associated with the subjective reports of drug reinforcement corroborating the relevance of drug-induced DA increases in the rewarding effects of drugs in humans. During withdrawal they have shown significant reductions in DA D2 receptors and in DA release in drug abusers. They have supported the original RDS concept [27] by postulating that this hypodopaminergic state would result in a decreased sensitivity to natural reinforcers, perpetuating the use of the drug as a means to compensate for this deficit and contributing to the anhedonia and dysphoria seen during withdrawal. Because the D2 reductions are associated with decreased activity in the anterior cingulate gyrus and in the orbitofrontal cortex they postulate that this is one of the mechanisms by which DA disruption leads to compulsive drug administration and the lack of control over drug intake in the drug-addicted individual. This is supported by studies showing that during craving these frontal regions become hyperactive in proportion to the intensity of the craving. Therefore, Volkow et al [58] postulate that dopamine contributes to addiction by disrupting the frontal cortical circuits that regulate motivation, drive, and self-control.

## Linking attention deficit disorder with obesity and dopamine

It is noteworthy that our laboratory has proposed that Attention Deficit Disorder (ADHD) is a subtype of RDS, having dopaminergic allelic associations among other deficit genes. In fact, being carriers of specific polymorphisms of the dopaminergic system places these individuals, both children and adults, at high risk for RDS behaviors (i.e. Substance Use Disorder [SUD] etc.) [59,60]. The linking of ADHD and obesity via a dopaminergic mechanism has also been proposed by others [61,62]. There is strong evidence indicating that dopamine dysregulation is very important in the pathophysiology of ADHD, as well as in the mechanism of the therapeutic action of stimulant drugs. With regard to therapeutic implications, recent studies indicate that methylphenidate (MPH), a drug widely used for ADHD, reduced overall energy intake with a selective reduction in dietary fat [61]. The findings are consistent with a reward deficiency model [34] of obesity whereby low brain dopamine predicts overeating and obesity, and administering agents that increase dopamine results in reduced feeding behavior. The obesity epidemic has focused attention on obesity's health consequences beyond cardio-vascular disease and diabetes. Current findings link both obesity and ADHD to the dopamine system and implicate dopamine genes in body weight, eating, and ADHD, among others. Detailed consideration suggests that dopaminergic changes in the prefrontal cortex among individuals with the ADHD subtype Attention Deficit Disorder (ADD) may increase their risk for obesity. Thus, individuals and populations with a high prevalence of hypodopaminergic genes may experience higher rates of obesity in the presence of abundant food [62]. From an evolutionary perspective, Campbell and Eisenberg [62] suggest that alterations in the dopamine system appear to affect a wide range of behavioral phenotypes. They suggest that recent evolutionary changes in the dopamine receptor genes selected to increase cognitive and behavioral flexibility may now be associated with attention problems and increased food consumption in an obesity gene environment.

With this said we must consider these results with caution, especially in terms of in vivo studies by Chen et al [63] showing a down-regulation of D2 receptor density following a 6 day infusion of the D2 agonist quinpirole [64]. Interestingly, continuous infusion of quinpirole caused a significant down-regulation of striatal D2 dopamine receptors without significantly changing the density of D1 receptors. This was accompanied by a decrease in the level of D2 receptor messenger RNA in the striatum as measured by northern blotting. The down-regulation of dopamine receptors was selective for D2 dopamine receptors. Moreover, continuous treatment with quinpirole resulted in a significant increase in striatal mu opioid receptor levels without significant change in the delta opioid receptors. This treatment also induced a significant decrease in proenkephalin messenger RNA in the striatum. Taken together, these results suggest that the down-regulation of D2 dopamine receptor and D2 receptor messenger RNA is the result of the persistent stimulation of D2 receptors and that the up-regulation of mu opioid receptors may be a compensatory response to a decreased biosynthesis of enkephalin. While this appears at first sight to contradict our suggestion, we theorize that the difference in continuous stimulation by a slow (more physiological and natural) release of DA, as proposed herein, will result in a proliferation in D2 receptors as seen in the in vitro studies [28,29] and documented by the consistent anti-craving effects observed in clinical trials [18,34-49].

It is noteworthy that diminished DA receptors are not inevitably associated with depression or addictive behaviors. In fact, while the lower incidence of Parkinson's disease (PD) among smokers may be explained by a protective effect of cigarette smoke, or by a tendency to avoid addictive behaviors among future PD cases, this does not hold true for alcoholism. Hernan et al [64] conducted an indirect test of the latter hypothesis by comparing the incidence of PD between alcoholics and nonalcoholics in the General Practice Research Database of the United Kingdom. Their case-control study included 1,019 cases and 10,123 matched controls. Overall, they did not find a lower incidence of PD among alcoholics compared with nonalcoholics (odds ratio: 1.09; 95 % CI: 0.67, 1.78). However, the contrary made be true. In Parkinson's disease, dopamine dysregulation syndrome (DDS) is characterized by severe dopamine addiction and behavioral disorders such as manic psychosis, hypersexuality, pathological gambling, and mood swings or Reward Deficiency Syndrome, as reported by Linazaroso et al [65]. In this regard, Witjas et al [66] describe the case of 2 young parkinsonian patients suffering from disabling motor fluctuations and dyskinesia associated with severe DDS. In addition to alleviating the motor disability in both patients, subthalamic nucleus (STN) deep brain stimulation greatly reduced the behavioral disorders as well as completely abolishing the addiction to dopaminergic treatment. According to the authors [66], dopaminergic addiction in patients with Parkinson's disease therefore does not constitute an obstacle to high-frequency STN stimulation, and this treatment may even cure the addiction. These findings related to Parkinson's disease partly support our proposal herein.

There is an abundance of studies showing that acute blockade of DA receptors will result in an attenuation of substance seeking as in the case observed for the Cannabinoid CB1 receptor antagonist, Rimonabant, which neuronal blocks DA-release [67]. This and other work has prompted Berridge [68] to rethink the role of DA as a so called "well-being substance". According to Berridge there are three competing explanatory categories: 'liking,' learning, and 'wanting.' Does dopamine mostly mediate the hedonic impact of reward ('liking')? Does it instead meadiate learned predictions of future reward, and stamp in associative links (learning)? Or does dopamine motivate the pursuit of rewards by attributing incentive salience to reward-related stimuli ('wanting')? In this regard, recent evidence indicates that dopamine is not needed for new learning, and is not sufficient to mediate learning directly by causing teaching or prediction signals. By contrast, growing evidence indicates that dopamine does contribute causally to incentive salience. Dopamine appears necessary for normal 'wanting', and dopamine activation can be sufficient to enhance cue-triggered incentive salience. Drugs of abuse that promote dopamine signals short-circuit and sensitize the dynamic mesolimbic mechanisms that evolved to attribute incentive salience to rewards. Such drugs interact with incentive salience integrations of Pavlovian associative information with physiological state signals. In short, dopamine's contribution appears to be chiefly to cause 'wanting' for hedonic rewards, more than 'liking' or learning for those rewards. Interestingly, Alcaro et al [69] agree with Berridge's view by suggesting that the rewarding properties of drugs of abuse are, in part, caused by the activation of the "SEEKING" disposition, ranging from appetitive drive to persistent craving depending on the intensity of the affect. The implications of such a view for understanding addiction are considered, with particular emphasis on factors predisposing individuals to develop compulsive drug seeking behaviors. In our view this predisposition is genetic and involves among other candidate genes the DRD2 gene. One important example of hedonic "wanting" and or "SEEKING" predisposition involves polymorphisms of the DRD 2 gene [70]. Statistical analysis revealed a significant association between the DRD2 TaqI A genotypes and "Eros" (a loving style characterized by a tendency to develop intense emotional experiences based on physical attraction to the partner), thus supporting hedonism as a "wanting" or "SEEKING" phenomena. Exploiting this view one might argue that the "reward center" be simplified and termed "the well-being system".

## Summary

In brief, the site of the brain where one experiences feelings of well being is the mesolimbic system. This part of the brain has been termed the "reward center". The chemical messages include serotonin, enkephalins, GABA and dopamine, all working in concert to provide a net release of DA at the NAc (a region in the mesolimbic system). It is well known that genes control the synthesis, vesicular storage, metabolism, receptor formation and catabolism of neurotransmitters. The polymorphic versions of these genes have certain variations, which could lead to an impairment of the neurochemical events involved in the neuronal release of DA. The cascade of these neuronal events has been termed "Brain Reward Cascade". A breakdown of this cascade will ultimately lead to a dysregulation and dysfunction of DA. Since DA has been established as the "pleasure molecule" and the "anti-stress molecule," any reduction in function could lead to reward deficiency and resultant aberrant substance seeking behavior. Our physiology is motivationally programmed to drink, eat, have sex and desire pleasurable experiences. Impairment of the mechanisms involved in these natural processes leads to multiple impulsive, compulsive and addictive behaviors governed by genetic polymorphic antecedents. While there are a plethora of genetic variations at the level of mesolimbic activity, polymorphisms of the serotonergic-2A receptor (5-HTT2a), dopamine D2 receptor (DRD2) and catechol-o-methyl-transferase (COMT) genes predispose individuals to excessive cravings and resultant aberrant behaviors. An umbrella term to describe common genetic antecedents of multiple impulsive, compulsive and addictive behaviors is RDS. Individuals possessing a paucity of serotonergic and/or dopaminergic receptors and a increased rate of synaptic DA catabolism due to a high catabolic genotype of the COMT gene are predisposed to self-medicating with any substance or behavior that will activate DA release, including alcohol, opiates, psychostimulants, nicotine, gambling, sex, and even excessive internet gaming, among others. Acute utilization of these substances induces a feeling of well being. But unfortunately, sustained and prolonged abuse leads to a toxic pseudo feeling of well being resulting in tolerance and disease or discomfort. Thus, low DA receptor levels consequent on carrying the DRD2 A1 allelic genotype result in excessive cravings and consequential behavior, whereas normal or high DA receptors levels result in low craving-induced behavior. In terms of preventing substance abuse, one goal would be to induce a proliferation of DA D2 receptors in genetically prone individuals. Experiments in vitro have shown that constant stimulation of the DA receptor system via a known D2 agonist results in significant proliferation of D2 receptors in spite of genetic antecedents. In essence, D2 receptor stimulation signals negative feedback mechanisms in the mesolimbic system to induce mRNA expression, causing proliferation of D2 receptors. This molecular finding serves as the basis for inducing DA release naturally, also causing the same induction of D2-directed mRNA and thus proliferation of D2 receptors in the human. This proliferation of D2 receptors will in turn induce the attenuation of craving behavior. In fact, as mentioned earlier, this has been proven with work showing DNA-directed overexpression (a form of gene therapy) of the DRD2 receptors and significant reduction in alcohol craving-induced behavior in animals [50]. Finally, utilizing long term dopaminergic activation will modify behaviors including Substance Use Disorders (SUD), Attention Deficit Hyperactivity Disorder (ADHD) and Obesity among other reward deficient aberrant behaviors. Support for the impulsive nature of individuals possessing dopaminergic gene variants is derived from a recent article suggesting that variants in the COMT gene predicts impulsive choice behavior, and may shed light on treatment targets [71].

A new but emerging concept provides a more comprehensive understanding of reward behaviors and the role of DA. In fact, interfering with accumbens DA appears partially to dissociate the process of primary reinforcement from processes regulating instrumental response initiation, maintenance and selection [72]. The fact that DA in the accumbens is involved with seeking maintenance suggests that activating the DA system over long periods of time rather than blocking DA receptors should result in attenuation of substance seeking behavior. This idea does not negate the important use of early detoxification whereby opioid receptors are blocked with Naloxone (Trexan/Rivera) [73], or DA activity is reduced with Acomposate [74] or with CB1 receptor blockers like Acomplia [75] among other similar approaches. It is noteworthy that assessment of 42 studies led to the conclusion that short-term administration of naltrexone significantly reduced the relapse rate, but was not associated with modification in the abstinence rate, suggesting the need for additional approaches [74].

We therefore suggest further that the biochemical and molecular changes that take place in dopaminergic and enkephalinergic systems following continuous neutraceutical treatment with dopamine agonists may underlie the mechanisms by which certain dopamine-mediated behaviors may be influenced. It is our intention to perform micro-dialysis studies showing that precursor amino acid therapy and enkephalinase inhibition induce DA release at the nucleus accumbens of both animals and humans, as well to perform additional clinical trials using nutrigenomic principles [76,77]. Finally our concept is supported by other work involving glutamate neurotoxicity. Preincubation with the D2 type dopamine agonists provides neuroprotection against glutamate neurotoxicity and the protective effects blocked by a D2 antagonist, indicating that D2 agonists provide protection mediated not only by the inhibition of dopamine turnover, but also via D2 type dopamine receptor [78]. While we caution interpretation our laboratory is encouraged that long -term dopaminergic agonistic therapy seems warranted.

## Competing interests

Kenneth Blum, Lonna Williams, B William Downs, and Roger Waite are officers of LifeGen Inc. and current stock holders. LifeGen, Inc. is the worldwide distributor of Synaptamine.™

## Authors' contributions

**KB **– Investigator and major contributor to writing of manuscript; **ALHCC **– Co-investigator and contributor to hypothesis; **TJHC **– Editorial contributions; **ERB **– Co-writer and editorial support; **SHB **– Literature search; **KC **– Editorial review; **BWD **– Major editor and co-writer; **RLW **– Contributor to clinical aspects of the commentary; **LW **– Editorial contributions; **TJP **– Statistical contributions and co-contributor to overall concept; **MK **– Editorial assistant and scientific review; **TP **– Editorial review; **DEC **– Contributor to scientific validity and editorial; **HT **– Editorial review; **JR **– Editorial review; **PR **– Editorial review; **MOB **– Contributor to writing manuscript and editorial and literature review.
